# A qualitative evaluation to explore the suitability, feasibility and acceptability of using a ‘celebration card’ intervention in primary care to improve the uptake of childhood vaccinations

**DOI:** 10.1186/s12875-016-0497-9

**Published:** 2016-07-30

**Authors:** Saumu Lwembe, Stuart A. Green, Nuttan Tanna, Jane Connor, Colin Valler, Ruth Barnes

**Affiliations:** 1 London School of Hygiene and Tropical Medicine, 15-17 Tavistock Place, London, W1H 9SH UK; 2 Centre for Healthcare Improvement and Research, Imperial College London, Chelsea and Westminster Hospital, Fulham Road, London, SW10 9NH UK; 3 Northwest London Hospitals NHS Trust, Watford Road, Harrow, HA1 3UJ UK; 4 London Borough of Hackney, Growth Boroughs Unit, 2 Hillman Street or 2nd Floor, Woolwich Centre, London, E8 1FB UK; 5 Royal Borough of Greenwich, Public Health and Wellbeing, 2nd Floor, Woolwich Centre, London, SE18 6HQ UK; 6 Sanofi Pasteur MSD, Mallards Reach, Bridge Avenue, Maidenhead, SL6 1QP UK

**Keywords:** Evaluation Studies, Vaccination, Primary Health Care, Communication, Social Marketing

## Abstract

**Background:**

Childhood vaccination remains a primary mechanism for reducing the burden of infectious disease. In the United Kingdom, as in many countries, a sustained effort is required to ensure that vaccination targets are met to afford protection to the whole population from vaccine preventable disease. The Celebrate and Protect programme is a collaborative partnership developed to improve the uptake of childhood vaccination across a number of boroughs within London through the use of a celebration card to encourage attendance for vaccination and enhance relationships between general practices and the parents/carers of children.

**Methods:**

This study was undertaken to assess the suitability, feasibility and acceptability of the Celebrate and Protect programme across nine boroughs in London. Data were collected either from telephone interviews (n = 24) or from focus groups (n = 31). A total of 55 key informants were included in the study, representing strategic, commissioning or policy leads, healthcare professionals and primary care teams delivering vaccinations and parents/carers of children under five.

**Results:**

The analysis of data identified that whilst parents/carers saw the celebration card positively this raised the issue of ‘vaccine hesitancy’ and the lack of information that parents/carers have to make informed decisions about vaccination. Similarly, healthcare professionals viewed the programme positively and felt that it was deliverable within existing resources although they raised wider questions about on-going sustainability and about quantitative data collection. In relation to the collaboration between primary care and a pharmaceutical company in developing the Celebrate and Protect programme, it was generally felt that, provided appropriate governance is in place, it was a pragmatic approach in which the benefits outweighed any perceived disadvantages.

**Discussion:**

The Celebrate and Protect programme was seen as an innovative collaborative programme to engage with parents and carers of children in order to improve relationships between service users and providers and subsequently increase vaccination uptake. The analysis demonstrates that that the celebration card is suitable for its purpose, acceptable to both healthcare professionals and to parents/carers of children and the Celebrate and Protect programme has been able to deliver its aims.

**Conclusion:**

Whilst the delivery of the ‘celebration card’ intervention in primary met its objectives there are some outstanding issues in terms of the sustainability of the initiative and the ability to demonstrate quantitative improvements in vaccination uptake rates.

## Background

### Vaccination in the UK

Vaccination is a public health intervention that has demonstrably reduced the global burden of infectious disease and which can, through comprehensive coverage, reduce health inequalities [1]. There are three main types of vaccination: childhood vaccination, adult vaccination and travel vaccination. Childhood vaccination results in individual immunisation against a range of diseases but also provides a level of indirect protection to the community when uptake is sufficiently high [2]. In the UK, childhood vaccinations are administered by General Practitioners (GPs), with uptake on a voluntary basis [3]. Timings of vaccinations have been set according the national schedule produced by the Department of Health (DH) informed by the age-specific risk for a disease, ability to respond to the vaccination and likelihood of developing complications [4].

Current estimates suggest that childhood vaccination coverage needs to reach 95 % to ensure herd immunity, a standard adopted by the World Health Organisation (WHO) [5]. Historically, immunisation uptake in London is variable and despite recent improvements many areas still perform below this target. With more than 350,000 children under the age of 5 eligible for vaccination in a single year (366,715 in 2010–11) ensuring 95 % of this population is vaccinated remains a logistical challenge. Data on the administration of vaccinations in primary care are recorded within the Child Health Information System [6]. These data are subsequently collated centrally to Cover of Vaccination Evaluated Rapidly (COVER), a national programme now managed by Public Health England (PHE), which gathers vaccination uptake data based on cohorts of children aged 12, 24 months and 5 years [7].

Despite the emphasis of both national and local public health programmes on ensuring the delivery of vaccines there is a wide geographical variation in uptake with some regions of England achieving less than 80 % coverage and other areas achieving 95 % or above [8]. In addition to the geographical variation many vaccination programmes demonstrate temporal variation. The most dramatic example has been with the Measles-Mumps-Rubella (MMR) vaccination, with a reduction to less than 80 % national coverage in 2003–4 associated with concerns about the safety of the vaccine which have since been discredited [9].

### Theoretical perspectives on vaccination uptake

Decision making by parents/carers plays an extremely important role in the immunisation process as the beliefs of parents/carers have been shown to strongly influence resulting vaccination of their child. These parents/carer beliefs and associated actions around vaccination can be classified as follows [10]:

- “Accepters”—those who believe in vaccination and actively seek to vaccinate their children;
- “Vaccine-hesitant”—those who accept vaccination but have significant concerns about vaccinating their children;
- “Late vaccinators”—those who purposely delay vaccinating or choose only some vaccines;
- “Rejecters”—those who completely reject vaccination.

It has been proposed that these typologies represent a continuum or spectrum of acceptance, from active demand for vaccines to complete refusal of all vaccines [11]. Benin et al. (2006) also identified a number of barriers to vaccination and some potential enablers or promoters [10]. Barriers included poor relationships with healthcare professionals resulting in a lack of trust, which may coincide with a strong trusting relationship with someone opposed to vaccination such as friends, family or other parents/carers. Of course this was often coupled with anxieties about the side effects of vaccines or a belief that herd immunity would protect their child. Some of the promoters of vaccination included good relationships with healthcare professionals that fostered trust and an open dialogue about vaccinations, and recognition that parents/carers may want to adhere to social/cultural norms by vaccinating. Those classified as “vaccine hesitant” or “late vaccinators” often have a broad range of reasons and rationales for their hesitancy to engage with vaccination, and an equally diverse range of external influences that may ‘nudge’ parents/carers along the continuum in either direction.

**Fig. 1:**
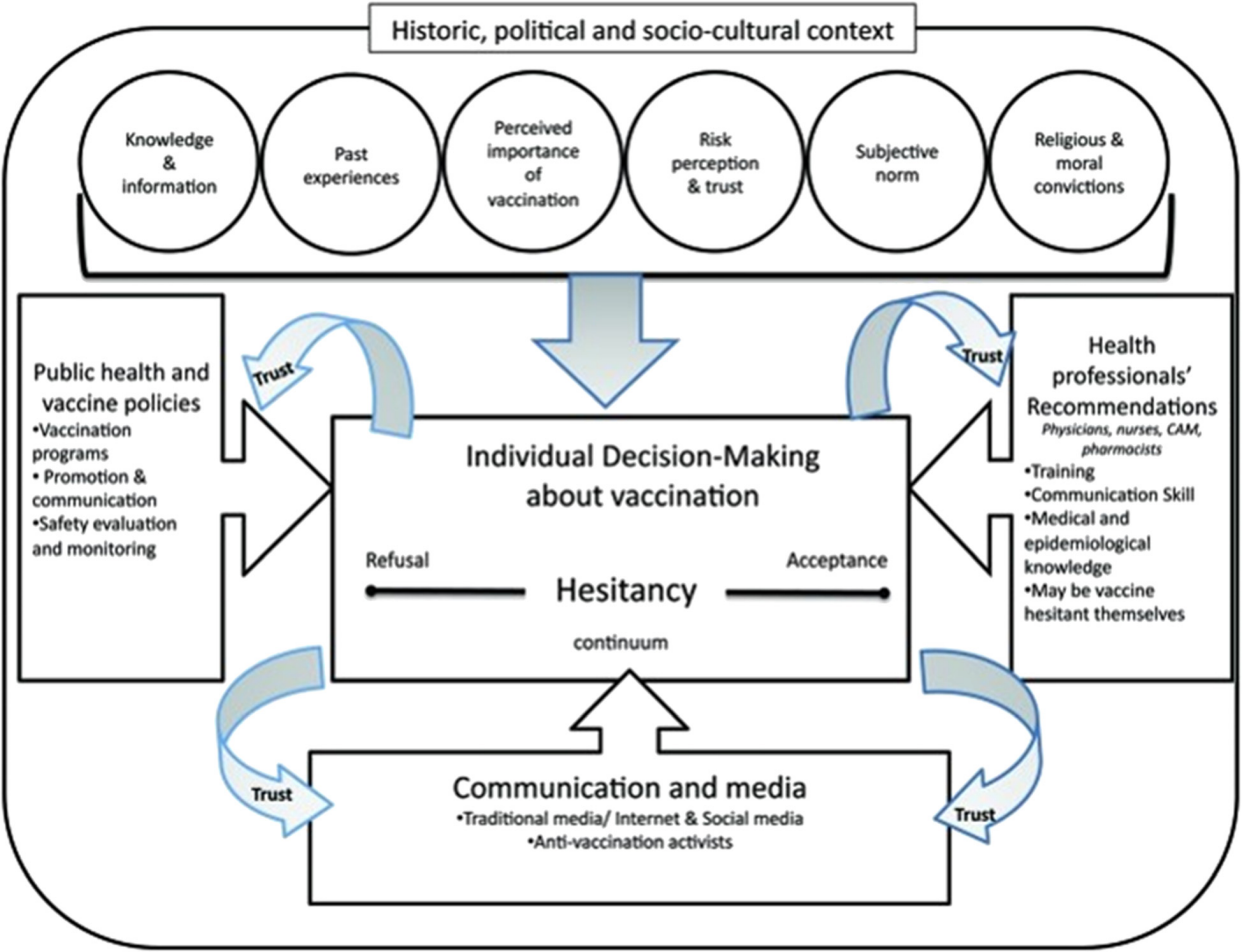
A conceptual model that encompasses many of the historical, political and socio-cultural factors that influence the position of parents/carers on the hesitancy continuum, all of which are underpinned by trust [11]

A conceptual model that encompasses many of the historical, political and socio-cultural factors that influence hesitancy, all of which are underpinned by trust influences, is shown in Fig. 1 [11].

### Interventions to improve vaccination uptake

A number of strategies have been developed that aim to target parents/carers to improve vaccination coverage, from simple strategies such as sending reminder letters/cards to more complex and costly strategies such as home visiting or involving school nurse teams [12, 13]. A recent systematic review demonstrated that ‘reminder and recall’ systems are an effective way of improving immunisation rates, although the development of locally appropriate systems is recommended [14]. Whilst initiatives included in the review have demonstrated some impact on vaccination, this is dependent on good systems of data collection/reporting and communication between systems and agencies to ensure timely and targeted activity. Furthermore, there is recognition that specific black and minority ethnic (BME) groups are often underrepresented in vaccination services, alongside those patients not registered with GPs [15].

It is clear that there is a need to develop effective programmes, based on theory, that can improve vaccination rates through engagement with parents and acting as an effective ‘reminder and recall’. In addition it may be necessary to remind patients why children need vaccination and as such these activities can be combined as a ‘call to action’.

This paper briefly describes an intervention that was developed as a ‘call to action’ to improve the uptake of childhood vaccinations in some areas of London. The paper also aims to provide a qualitative evaluation to assess the feasibility, suitability and acceptability of the delivery of the intervention by identifying specific barriers and facilitators to delivering the programme and to provide some suggestions for learning in future programmes.

## Methods

This section briefly describes the programme that was delivered but focuses on describing the methodological approach to undertaking a qualitative evaluation to assess the feasibility, suitability and acceptability of the programme from the perspectives of parents/carers, health care practitioners and policy makers.

### The celebrate and protect programme—a communication intervention

NHS Barking and Dagenham, a Primary Care Trust (PCT),^1^ in partnership with Sanofi Pasteur MSD (SPMSD), a vaccine manufacturer, developed the Celebrate and Protect programme in 2012. The initiative was part of a wider Olympic and Paralympic Host Borough health legacy programme linked to the London 2012 Olympic Games. The programme aimed to increase uptake of childhood vaccination by supplementing current General Practitioner (GP) practices’ current call/recall activities and through improved engagement between families and GP practices. The intervention consisted of a celebration card and immunisation schedule sent out by the GP practice staff to families of children before their vaccination was scheduled. The celebration card was intended to act as a ‘call to action’ for the parents/carers of children under five to attend an initial 6–8 week check (new-borns) and vaccination appointments (1 year olds and 4 year olds) with their GP practice.

The first wave of the programme was initiated in July 2012 in nine London PCTs: Barking & Dagenham, Bexley, Greenwich, Kensington & Chelsea, Hammersmith & Fulham, Newham, Tower Hamlets, Waltham Forest and Westminster. Part of the scope of the initial wave was to assess the feasibility and acceptability of the programme through engaging with a range of stakeholders and iterative development of the intervention to allow a tailored rollout across additional PCTs in London and possibly further afield. During the first wave, practices across the PCTs were contacted and asked to participate with the aim of engaging 50 % of practices. Across the nine PCTs, 66.3 % (n = 177) of GP practices were recruited, ranging from 42 to 100 % of practices within the PCTs.

A personalised celebration card and an information leaflet with a vaccination schedule was co-designed with parents/carers. Workshops were held with parents/carers of children to assess the response to the design of the cards, which included a central character and the inclusion of landmarks from east and central London associated with the Olympic and Paralympic games. In addition, the celebration cards included a statement on the back regarding partnership between the NHS and Sanofi Pasteur MSD.

Following training sessions for practice nurses and managers, the celebration cards were delivered to participating practices. The programme management team arranged for an appropriate number of celebration cards and pre-franked (second class postage) envelopes to be dispatched to the individual GP practices according to the number of registered children within each cohort, plus a 5 % uplift. The cards were subsequently sent out by the GP practice to parents/carers registered at the practice following the birth of a child or prior to the first or fourth birthday of a child registered at the practice. The card intended to celebrate the birth of a child or a child’s birthday and act as a ‘call to action’ for the parent/carers to contact the practice and book a health check or vaccination.

The card for new-borns included a message inviting parents/carers to make an appointment with the practice to come and discuss any questions they had about the baby’s health and for the baby to be examined, at which time it is usual for babies to receive their first set of vaccinations. Cards distributed within PCTs that had a universal tuberculosis (TB) vaccination programme (i.e. where the incidence of TB is greater than 40/100,000 population) included an additional message for parents/carers to make an appointment for TB vaccination [16].

Birthday cards for 1 year olds included a message that the child’s vaccinations were due and an invitation to contact the GP practice to make an appointment. Birthday cards sent to the 4 year olds also had a message to contact their GP practice to make an appointment. However, this card was only sent to those children who had not yet received their immunisations, so this card acted as a failsafe. The cards also contained information signposting parents/carers to the ‘Red Book’, the Personal Child Health Record which is a national standard health and development record given to parents/carers at a child’s birth, and www.immunisation.nhs.uk, along with an insert with information about the schedule of vaccination as recommended by the DH.

### Participants

Sample groups were identified to include stakeholders at three different levels (Table 1):

**Table 1:** Outline of participants included in the qualitative study

Group	Data collection	Grouping	Participants
1	Telephone interviews	Policy makers	Immunisation co-ordinators (*n* = 5), public health consultants (*n* = 3), senior public health managers (*n* = 5) & industry stakeholders (*n* = 2)
2	Telephone interviews	Practitioners	Participants included administrators (*n* = 2), managers (*n* = 4), GP (*n* = 1) and nursing staff (*n* = 2)
3	Focus Groups	Parents/carers of children under 5	Parents/carers (*n* = 31)

- *Group one*—policymakers (n = 15): this included strategic, commissioning or policy leads along with the programme management team, a purposive sample identified from the broader stakeholder group.
- *Group two*—practitioners (n = 9): primary care staff were recruited by canvassing 40 (23 %) GP practices involved in wave one via emails from the PCT immunisation co-ordinators.
- *Group three*—parents/carers of children under five (n = 31): participants were recruited via the PCT immunisation co-ordinators with the aim of identifying 2–3 participants from each PCT, although respondents were affiliated to just six of the nine PCTs involved in the Celebrate and Protect programme. Whilst the sample was not specifically designed to be representative the participants were of diverse ethnic and socio-economic backgrounds.

### Data collection

Semi-structured telephone interviews were undertaken with all participants in groups one and two by a member of the evaluation team. The rationale for this was simply pragmatic; to attempt to bring individuals together from across London is resource intensive. In addition, undertaking telephone interviews was preferable for the research participants as this provided a flexible mechanism for them to contribute where they may have declined involvement had they be asked to attend a meeting in person. Focus groups were selected as the most appropriate data collection methods for parents and carers to include as many views as possible but with limited available resources.

A total of three focus groups were conducted in three locations: east, south east and north west London between October 2012 and February 2013 with 3, 10 and 18 participants respectively. A member of the evaluation team facilitated the focus groups and a topic guide led discussions. Proceedings from the focus groups and interviews were audio-recorded where possible (with the consent of participants), transcribed by a project administrator and validated by two evaluation team members. Oral consent was requested and recorded from each participant for both telephone-interviews and focus groups.

### Analysis

Transcripts and field notes from all interviews and focus groups were analysed thematically by a member of the evaluation team using the Johnson and Sholes (2005) suitability, feasibility and acceptability framework [17]. *Suitability* is defined as the rationale for developing a particular strategy and whether this strategy supports the organisational or programme aims; *feasibility* relates to the availability of organisational or programmatic resources, skills and competencies to deliver a particular strategy; and *acceptability* describes the reaction of stakeholders and the likelihood of encountering potential organisational or programmatic risks [17]. The researcher that undertook the data collection (NT) is a female pharmacist with a PhD. The researchers that analysed the data are female (SL) and male (SG) and are public health practitioners and researchers, respectively, both with master degrees and completing their doctor of public health qualifications. All have performed research studies including focus group and interview in previous studies.

## Results

### Suitability

The Celebrate and Protect programme aimed to improve the uptake of immunisation services by parents/carers in order to increase vaccination coverage in those areas of London where the programme was delivered. It was also developed to encourage parents/carers receiving the celebration cards to proactively engage with their GP practices in scheduling vaccination for their children. Understanding how parents/carers perceived the cards and their intended action was important to validate the mechanism. Responses from the focus groups (and some providers) indicated that the participants’ perceptions of the celebration cards were more of a reminder than a ‘call to action’. This may be explained by the fact that some parents/carers may already have received a letter from their GP reminding them the time to immunise the children was due and the card, in this instance, could be seen as an additional reminder.

Participants in the focus groups were asked to comment on the content of the celebration cards, and whether sufficient information was presented to allow them to take the necessary action. Some participants felt not enough information was provided about why vaccination was necessary and the timing of steps that were needed to vaccinate their child. Many parents/carers felt that this information was necessary to allay their anxieties or fears where there were reservations regarding immunisation. This was countered somewhat by the inclusion of scheduling information provided on an insert to be included with the cards. Parents/carers felt the content was likely to be effective in prompting action amongst people not opposed to immunisation, although it would be less effective with those opposed to getting their children immunised.

*“…There’s nothing on here to say why you should have your baby immunised…” “…Quite dry information, it just gives you the name of the inoculation. I’m not a doctor… Haemophilus influenzae Type B, what does that protect my baby with…?”* (*Group 3-parents/carers).*

On the other hand, it was apparent that without including more direct information regarding what actions parents/carers need to take on receiving the card, there would be a risk of parents/carers perceiving the card as a good gesture from practices as opposed to a call for action.

*“…I think it’s a very good idea and it’s wonderful, however…I’d presume.... it’s just a card, a congratulation card…”* (*Group 3-parents/carers).*

Focus group participants made suggestions on how the cards could be improved in a way that could potentially sway the minds of those opposed to immunisation:

*“…put a little bit maybe on the back of there and say, well we could give hemacoccal [sic] against meningitis…” “…I think …explain what some of the diseases that immunisation is trying to protect against…”* (*Group 3-parents/carers).*

Participants felt adding a link to credible website would be invaluable:

*“…link, with the added recommendation that you can call your healthcare professional* via *telephone on the card....”.* Or add that *“…If you have any questions or concerns please come along or give us a ring…”* because *“…just think it gives leeway for people who feel a bit insecure and nervous, sort of isolated; it just makes it more robust…the information… it’s not just a reminder…you know, if you have any concerns, we’ll be happy to speak with you…”* (*Group 3-parents/carers).*

And as one of the parents/carers mentioned:

*“… although I always had the intention to keep up with all the immunisation, I was always a little bit sceptical and worried about immunisation…I would like to be more informed about the [unclear] side effects of the immunisation…”* (*Group 3-parents/carers).*

Aside from comments on the contents of the card the focus groups discussions also addressed the desirability or potential demand for the cards, indicating that if they missed out on the celebration card many of the parents/carers would feel offended or excluded. From the provider perspective, the cards also seemed to be very popular, evidenced by the ability to recruit additional PCTs for subsequent waves of the Celebrate and Protect programme: ‘[Celebrate and Protect] *Impressive…’ …12 boroughs* [PCTs] *signed up.... 175 practices registered…definitely a starting point…can only go up.... parents more aware of imm[unisations]…”* (*Group 1-policymakers).*

Also retention rates of the PCTs in subsequent phases were suggestive of the perceived effectiveness and popularity of the programme with two thirds continuing to be involved in the second phase, which covered an additional three PCTs. Nevertheless, responses from some strategic leads indicated that at a practice level there were notable variations in engagement:

*“…there are practice variances - with some with total buy in and some that do not want to know/do not want any extra workload”* (*Group 1-policymakers).*

Understanding the perceived added value of the Celebrate and Protect programme was also important to encourage future engagement. Some participants, especially from the strategic leads and providers groups expressed the opinion that celebration cards played a necessary role in helping to communicate an issue that is otherwise delicate or difficult to broker with some parents/carers. They felt that celebration cards offered a suitable alternative:

*“…Celebrate and Protect supports communication…it makes things easier…”* Moreover, in their experiences, *“…mothers are reluctant to go to practices (for vaccination) unless invited as they feel surgeries are busy”* and *“do not like letters that sound threatening”* (*Group 1-policymakers).*

Additionally some providers felt that the Celebrate and Protect programme added value to the call/recall system and was of particular value to practices without a robust call/recall system:

*“…we were in bottom 10, since Celebrate and Protect, we are now in top 10…” (Group 2-practitioners).*

Practices that had an established call/recall system also noted the added value:

*“… get [Celebrate and Protect] people up to date with vaccinations… going fine, actually…asked a couple of parents.... those asked said…reminded me to come…very happy…”“…Celebrate and Protect good for ‘impetus’ to start thinking about immunisation but need other basic processes in place, e.g. good call and recall system in practice and at PCT level; follow ups of defaulters; need for more ‘support processes…”* (*Group 2-practitioners).*

Some providers however did not perceive the Celebrate and Protect programme as adjunct to a call/recall system but a substitute:

*“…Celebrate and Protect.... birthday cards have lessened my workload…don’t have to make phone calls.... surgery does not have to pay for postage.... reduced workload as do not have to speak to address concerns…”* (*Group 2-practitioners).*

Some parents/carers who had good existing relationships with their practices didn’t see any added value to the card:

*“…Well I…I don’t really need because I have …the Red Book…my doctor rang me and sent a text, so I get reminded all the time”. “I still think it’s a really good idea… (but)… a letter would be better I wouldn’t need a pretty card”* (*Group 3-parents/carers).*

Others who didn’t have existing relationships with their practice felt that the card would be more helpful but were keen to stress that the programme should not be an alternative to the current call/recall system:

*“… I think you can’t take away from people, like face to face or call…”“…and if then they (mothers) say no then they can actually talk to them about the reason, so you’re addressing…any other issues that they might have…”* (*Group 3-parents/carers).*

### Feasibility

Responses from the strategic leads indicated that the Celebrate and Protect programme was made possible by the involvement of immunisation leads linked to PCTs that had historically established trust with GP practices. The leads were encouraged to facilitate implementation through communication and provision of training and training materials to GP practices.

*“… (Leads) were able to secure commitment from GP practices - as they have already established trust/good relationship with GP practices, so much easier to get GP buy-in to the project…”* (*Group 1-policymakers).*

Some providers explained that implementation of the Celebrate and Protect programme didn’t require additional resources:

“[we did] *not needed any support from lead PCT for Celebrate and Protect all very straightforward…with information pack…useful guide on how to do labels....” “.... working fine…not much extra work per month....”* (*Group 2-practitioners).*

If anything, some practices felt the Celebrate and Protect programme gave them the additional resources to support their work:

*“…before that (Celebrate and Protect) it was just me … Celebrate and Protect added boost…” “…it’s not a big job to do…an hour a month…”* (*Group 2-practitioners).*

Strategically however, there was a perceived need to secure commitment from an organisation to take a lead role (e.g. NHS London), because, thus far, the programme was largely driven by individuals, with a risk to its sustainability in the face of the imminent NHS structural changes.

*“…Celebrate and Protect is driven by power of personality, not supported by system....”* (*Group 1-policymakers).*

Many of the strategic leads felt that the Celebrate and Protect programme was a low cost initiative with an implementation strategy that could allow roll-out across other areas on both a local and national scale. Participants felt the programme was financially viable, especially for practices falling short of their target of 95 % coverage.

Particular challenges around the ability to develop the evidence of the impact of the Celebrate and Protect programme were clear from responses from providers and strategic leads. Some respondents cited historical problems around the lack of robust performance management data systems, with inconsistencies in how data were captured and communicated across the different data systems used in the immunisation programme. Some felt that there were on-going inconsistencies on how data were recorded and that these issues may make it difficult to demonstrate the effect of the Celebrate and Protect programme.

However, direct feedback from some of the providers suggested that the Celebrate and Protect programme had improved uptake of immunisation:

*“… we are doing a lot better than we were…imms uptake has improved…no negative feedback from patients or staff…” “.... percentage of children coming in has risen … imms uptake [has increased].... our take up for 6 week check…good…anyway…”* (*Group 2-practitioners)*

Whilst the responses were positive in terms of the perceived increase in immunisation it would be difficult to attribute causality solely to the celebration cards. There were of course also challenges to implementation that were identified, especially relating to staffing:

*“…Cards were (in a box) waiting for me when I returned from Maternity leave” “…difficult in our practice…” “…takes time…writing names and address on envelope.... do not generate labels…”* (*Group 2-practitioners).*

Some operational challenges cited included the time taken to generate address labels. It was also felt that the Celebrate and Protect programme needed a lot of attention to detail for fear of missing any children:

*“…fantastic but…time consuming* [especially] *mail merge…have to ensure search is correct’.... one*[issue]....*’can miss new registrants in between monthly searches.... uses Open Exeter…” “…takes time…writing names and address on envelope.... do not generate labels…”* (*Group 2-practitioners).*

Some providers suggested expanding the remit of the Celebrate and Protect programme to include those children and their parents/carers not registered with a GP through engaging with health visitors and liaising with maternity units to co-ordinate from birth:

“…(Celebrate and Protect) *doesn’t cover new parents/carers … they do not see us … see health visitor…health visitors remind them but [the] call has not come from [the] surgery so mothers forget…”* “*some parents take time to register their new-born”* (*Group 2-practitioners).*

### Acceptability

Whilst the idea of receiving celebration cards was rated highly by all categories of respondents, less than 10 % of the parents/carers attending the focus group had received one. Much of their response to the cards was framed as a comparison to more traditional call/recall mechanisms, especially practice letters.

*“…do you see what I mean? You need to get your child to the clinic. You need to get them immunised. This* [celebration card] *is like; it is more of a positive reinforcement. The letter is more; you have been told off. This is more like… it is colourful…” “…good idea… a good reminder…because you have a little baby, sleepless nights, sometimes you might forget to make an appointment”, “Oh, even I missed the first MMR when he was two because I forgot, I’m involved in health and I forgot to get him immunised…* (the card would have) *without a doubt (helped)…”* (*Group 3-parents/carers).*

This was echoed by the providers who felt it was more acceptable to send celebration cards, as it would enhance the call/recall systems and improve relationships with patients.

One particular aspect of the Celebrate and Protect programme—working closely with a pharmaceutical company—was seen as particularly novel but it was recognised that this could have some inherent risks. All categories of respondents expressed opinions related to the decision to work in collaboration with a pharmaceutical company and most responses were positive:

*“…personally.... supportive of this stance.... embrace new ways of working.... learn from partners....”*(*Group 1-policymakers); “couple of years ago…probably would have had more reservations…but now as long as ethical issues are covered as required by DH policy document…we need to get used to working with private providers” “Personally ‘don’t have an issue”,* because *the “reputation of pharmaceuticals is changing”…* (*Group 2-practitioners).*

The financial benefits of pharmaceutical industry involvement were recognised by some:

*“…growing reality…cannot afford purely a PH project as high costs.... three times costs for distribution and procurement” “…in the new world …you want to continue [working] with Sanofi.... they will make a corporate social responsibility contribution....”* (*Group 2-practitioners).*

Others expressed concerns about how this was represented:

*“…uncomfortable territory for some…”*(*Group 2-practitioners); “…we.... met a lot of resistance … whether it was appropriate to work with a pharmaceutical company…[despite] DH guidelines [to].... support partnership working with industry….* [there is] *local level resistance to engaging with this philosophy…”* (*Group 1-policymakers).*

Asked on their views about the NHS working with a pharmaceutical company, parents/carers were generally accepting of the concept provided that necessary approvals and authorisations were undertaken:

“*…If the NHS have approved, then I am okay with it…”* (*Group 3-parents/carers).*

Others however were quite sceptical in view of previously televised broadcasts on unethical activities by some pharmaceutical companies:

*“…I saw on the telly about price fixing with pharmaceutical companies, where they offer GPs incentives to prescribe their product....”* (*Group 3-parents/carers).*

Despite this suggestion, the aim of the Celebrate and Protect programme was primarily to increase vaccination and to improve relationships between parents/carers and primary care practices. Parents/carers identified with this aspect of the programme:

*“… (it would make one) feel warmer towards your GP surgery”,* “…*If you received a card from GP you would feel cared for…”* (*Group 3-parents/carers)*

However, this was not shared by all:

*“No. I don’t think it would make a difference for me because my practice is always grumpy. I don’t think it’ll change that”* (*Group 3-parents/carers).*

## Discussion

The Celebrate and Protect programme has demonstrated innovative partnership working bringing together strategic leads from the NHS and local government alongside partners from the pharmaceutical industry, Sanofi Pasteur MSD. The celebration card, which formed the main intervention delivered within the programme, was co-designed with parents/carers, who were able to influence the design and content of the card, providing some insight about why parents/carers may or may not choose to have their children immunised.

Whilst this analysis did aim to assess the effectiveness of the Celebrate and protect programme in improving vaccination rates amongst different typologies of parents/carers, the programme is unlikely to have any significant effect on vaccine ‘rejecters’ and most likely to impact ‘vaccine hesitant’ parents/carers [10]. Furthermore, whilst the evaluation did not specifically assess the impact of ethnic and cultural background, there was some attempt at cultural inclusivity with information being provided in a number of languages, which again may support inclusion of BME communities, although makes no explicit attempts to address some of the more complex underlying issues [15]. In addition, there was a strong association between the cards and the city of London, through the use of identifiable landmarks, and many of the images were representative of the links with the London 2012 Olympics, which could be seen as a globally inclusive event. Furthermore inner city areas with high density, deprived populations have been shown to have particularly low rates of MMR uptake and have been identified as requiring specific interventions to improve vaccination uptake.

### Suitability, feasibility and acceptability

The Feasibility, Suitability and Acceptability framework is used to assess the relative success or failure of a strategic option.

Following the qualitative evaluation, it would seem that the Celebrate and Protect programme could be judged as a low risk and low cost strategy that could be delivered feasibly within the resources and competencies that were available during wave one, although significant organisational and structural changes have occurred since then. The specific intervention (i.e. the celebration card and vaccine schedule) used within the programme has been shown to be suitable for achieving the objectives of the programme, although demonstrating the effectiveness of the intervention in terms of its impact on vaccination uptake remains a challenge. Whilst the intervention has been shown to be acceptable both to those delivering it (i.e. primary care staff) and to the intended recipients (i.e. parents/carers of children under the age of 5), a number of recommendations were identified from the focus groups that resulted in changes to the intervention for subsequent waves. This iterative development demonstrates the flexibility and adaptability of the celebration card.

Acceptability of the programme at a strategic level was demonstrated throughout the evaluation, although due to the structural reorganisation of the NHS and changes in the roles and responsibilities of those initially involved in the programme, it should be recognised that there are important risks in terms of managing the relationships crucial to the feasibility of the Celebrate and Protect programme in the longer term.

### Collaboration with industry

Collaboration with ‘industry’ can provide economies of scale along with strategic expertise and an in-depth understanding of logistics, which were fundamental to the delivery of the Celebrate and Protect programme. In addition it was observed that an important role of the industry partner, especially during the time of NHS transition experienced during the programme, was the consistency and continuity offered to the programme. As the Celebrate and Protect programme was initiated in conjunction with the industry partner, a unique collaborative framework was provided through which many different organisations voluntarily co-operated to develop a ‘bottom-up’ potential solution to an intractable problem. This benefit of course needs to be measured against any potential conflicts of interest that may be represented by such collaborations. In this case, as childhood vaccine is procured nationally through a tendering process involving multiple pharmaceutical companies, there appears to be no immediate conflict of interest in working with SPMSD given that the partnership abides by the DH/Association of British Pharmaceutical Industries Framework on partnership with pharmaceutical companies [18].

### Limitations

Whilst the sampling strategy identified a broad range of parents/carers, for pragmatic reasons it did not specifically target the intended beneficiaries of the Celebrate and Protect programme in that the parents/carers attending the focus groups were not necessarily registered with GP practices participating in the first wave of the programme. In addition, the recruitment of primary care staff coincided with a busy influenza vaccination season resulting in a lower than expected participation.

## Conclusion

This paper reports on the findings of a study to assess the feasibility, suitability and acceptability of the Celebrate and Protect programme. The programme is innovative in a number of ways, having been established collaboratively by a range of partners including PCTs, GP practices, local authorities and a pharmaceutical company producing childhood vaccines. It uses a novel method (the celebration card) to engage with parents and carers of children in order to increase vaccination uptake and to improve relationships between service users and providers. The card was designed with input from all the relevant stakeholders, including parents/carers, and the programme was developed iteratively, with on-going review and evaluation so that potential improvements could be tested rapidly and incorporated without delay.

The analysis presented here focuses on the suitability, acceptability and feasibility of the programme, drawing on qualitative data collected through interviews and focus groups as part of a wider evaluation process, which also involved a quantitative analysis of vaccination uptake rates. The analysis identified that many of the factors that have been proposed in the conceptual model of vaccine acceptance/rejection are indeed important to parents/carers [11].

From this qualitative analysis, it is clear that the celebration card is suitable for purpose and acceptable both to healthcare professionals and to parents/carers of children. In terms of feasibility, the Celebrate and Protect programme has thus far been able to deliver its process aims (i.e. recruitment of practices and delivery of the celebration cards) within the allocated resources although several issues of on-going sustainability were raised, mainly in relation to the changing primary care infrastructure. In addition, the question of whether the very positive views expressed about the Celebrate and Protect programme can be linked to more quantitative outputs and outcomes (such as vaccination uptake rates) was not within the scope of this analysis but remains an important line of further inquiry.

## Abbreviations

BME, black and minority ethnic; COVER, Cover of Vaccination Evaluated Rapidly; DH, Department of Health; GP, General Practitioner; MMR, measles, mumps and rubella; PCT, Primary Care Trust; PHE, Public Health England; SPMSD, Sanofi Pasteur MSD; TB, tuberculosis; WHO, World Health Organisation

## Footnotes

1 As a result of the Health and Social Care Act 2012, Primary Care Trusts ceased to exist on 31 March 2013 and were replaced by Clinical Commissioning Groups (CCGs). In London the PCTs were broadly coterminous with local authority boroughs and were the geographical unit for which immunisation rates were recorded.
